# Surgical management of a large neurofibroma in the thenar Region: A case report

**DOI:** 10.1016/j.ijscr.2024.110742

**Published:** 2024-12-17

**Authors:** ELMehdi Bachkira, Imad Jadib, Amine Errajragi, Ahmed Ghannam, Elkassimi Charafeddine, Abdeljebbar Messoudi, Mohamed Rafai

**Affiliations:** Department of Orthopedics and Trauma-Surgery P32, University Hospital Center IBN Rochd, Casablanca, Morocco

**Keywords:** Neurofibroma, Thenar region, Hand tumor, Median nerve, Surgical excision, Case report

## Abstract

**Introduction:**

Neurofibromas are rare benign tumors of peripheral nerve sheaths, and hand involvement is particularly uncommon. This case report presents a large neurofibroma located in the thenar region, a critical area for thumb opposition and hand dexterity, posing unique surgical challenges.

**Presentation of case:**

A 23-year-old female presented with a 3-year history of a progressively enlarging mass in the thenar region of the right hand, accompanied by nocturnal pain but no neurological deficits. MRI revealed a well-circumscribed, multilobulated tumor measuring 54 × 55 × 33.4 mm. Surgical excision was successfully performed while preserving the median nerve. Postoperative recovery was assessed using the QuickDASH questionnaire, yielding a score of 6, indicative of excellent functional outcomes. Histopathology confirmed a benign neurofibroma.

**Clinical discussion:**

The large size and rare location of this tumor, combined with the absence of neurofibromatosis type 1, make this case noteworthy. Early diagnosis and meticulous surgical planning enabled complete excision while preserving critical structures, resulting in full functional recovery with no recurrence after 18 months.

**Conclusion:**

This case underscores the importance of individualized surgical strategies for managing neurofibromas in functionally critical areas. While excellent long-term outcomes were achieved in this instance, further studies are needed to validate these findings and refine management approaches.

## Highlights

• Rare large neurofibroma located in the thenar region, essential for thumb opposition and hand dexterity.
• Tumor caused rapid functional impairment, underscoring the significance of its location.
• Successful surgical excision preserved the median nerve, with full functional recovery after 18 months.
• Absence of neurofibromatosis type 1 (NF1) adds diagnostic complexity, highlighting the need for early, tailored intervention.

## 1 Introduction

Neurofibromas represent benign proliferations of the peripheral nerve sheaths, known for their diverse clinical manifestations, progression, and outcomes. These tumors typically exhibit a complex cellular composition, blending Schwann cells, fibroblasts, and perineural-like cells, all embedded in a myxoid matrix. A distinct feature of neurofibromas, differentiating them from schwannomas, is the presence of intratumoral nerve fibers, a characteristic that defines their unique histopathological profile [1,2]. Clinically and histologically, neurofibromas are categorized into various subtypes, each presenting its own diagnostic and therapeutic challenges [1,2].

While neurofibromas can occur throughout the body, their manifestation in the hand is particularly rare and clinically significant due to the intricate network of nerves, tendons, and muscles. Managing such a condition requires careful surgical planning to preserve the delicate motor and sensory functions of the hand. Large neurofibromas in this region present even greater challenges, as their size and proximity to critical structures raise the stakes for potential postoperative complications.

In this case report, we present a unique instance of a massive neurofibroma of the soft tissues in the hand, a rare and instructive case that highlights both the diagnostic complexity and the surgical intricacies involved in treating such a tumor. By documenting this case, we aim to contribute valuable insights to the limited literature on hand neurofibromas, emphasizing the importance of meticulous preoperative planning and the need for a precise, nerve-sparing surgical approach to optimize functional outcomes.

We obtained informed consent from the patient to use their information and images for the publication of this manuscript. This case report has been written in accordance with the SCARE 2023 guidelines [3].

## 2 Case presentation

We report the case of a 23-year-old female patient who presented with a swelling on the palmar surface of the right hand, progressively increasing in size over the course of three years, without any systemic symptoms. The patient reported nocturnal pain radiating to the thumb, index, and middle fingers, but without any neurological deficits. She also experienced functional discomfort during grasping movements.

**Fig. 1:**
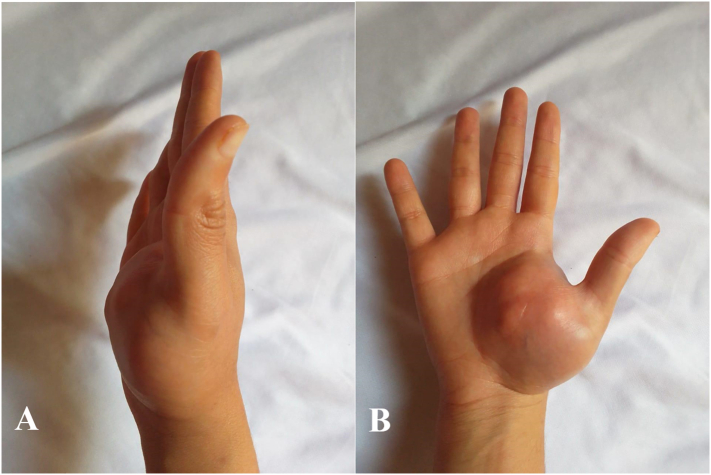
(A + B): Pre-operative Clinical Presentation: Showing a visible mass in the thenar region.

Clinically, the mass was located in the thenar region of the right hand, measuring 7 cm at its longest axis. It was fixed to the deeper planes but mobile relative to the superficial ones. The mass was firm in consistency, and the overlying skin appeared normal, with no telangiectasias, collateral circulation, or signs of inflammation (Fig. 1). Lymph node areas were free from any abnormalities. General examination did not reveal other masses or café-au-lait spots suggestive of neurofibromatosis type 1.

**Fig. 2:**
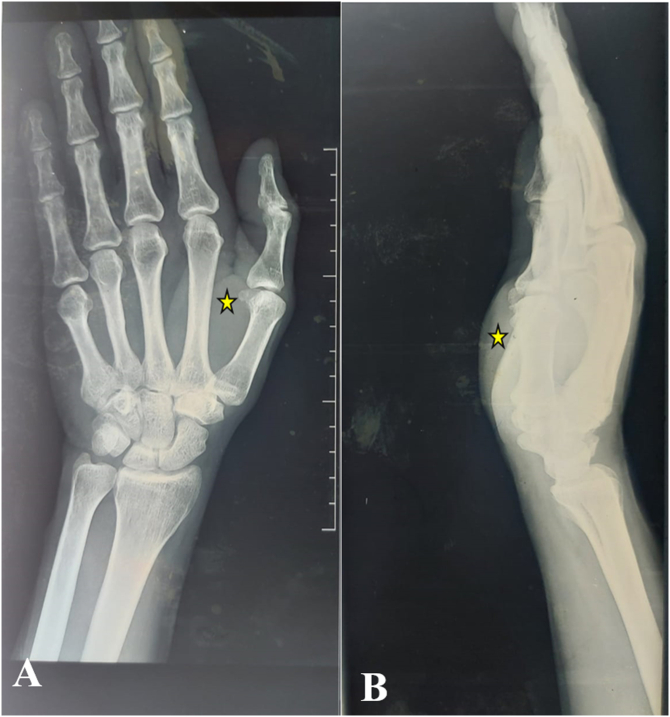
(A + B): A standard left hand radiograph revealed increased soft tissue density (), with no evidence of calcifications or associated bone lesions.

**Fig. 3:**
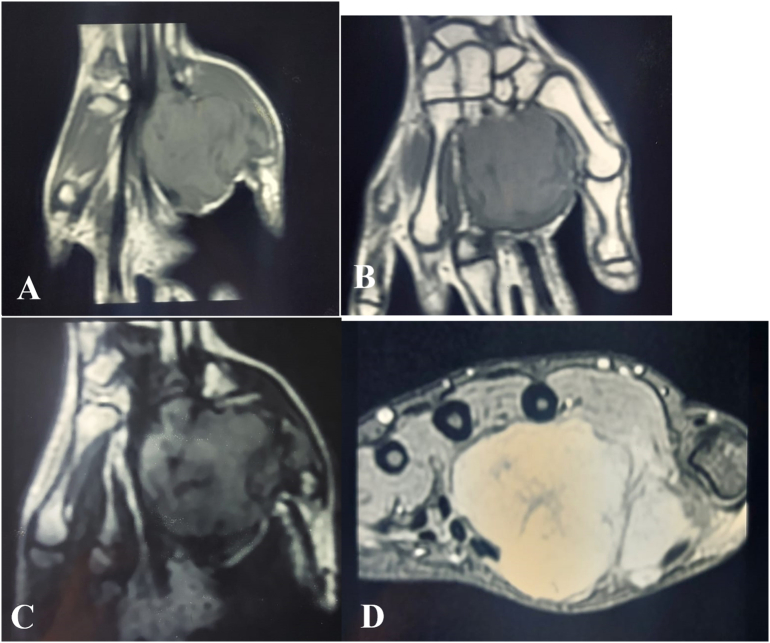
(A + B + C + D): MRI of the right hand revealed a well-circumscribed mass in the thenar region,

A standard radiograph of the hand showed increased soft tissue density without calcifications or associated bone lesions (Fig. 2). MRI of the right hand revealed a well-circumscribed mass in the thenar region, measuring 54 × 55 × 33.4 mm, showing intermediate signal intensity on T1- and T2-weighted images, and a high STIR signal, with intense and heterogeneous enhancement following gadolinium injection (Fig. 3). Preoperative electroneuromyography was performed to evaluate the median nerve, revealing normal conduction with no latency or evidence of conduction abnormalities.

**Fig. 4:**
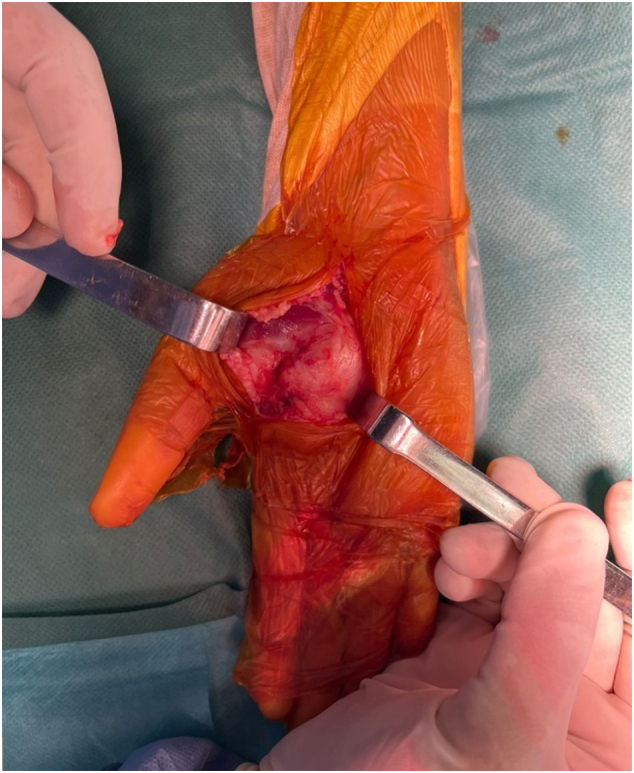
Surgical approach involved a longitudinal incision along the thumb opposition crease.

**Fig. 5:**
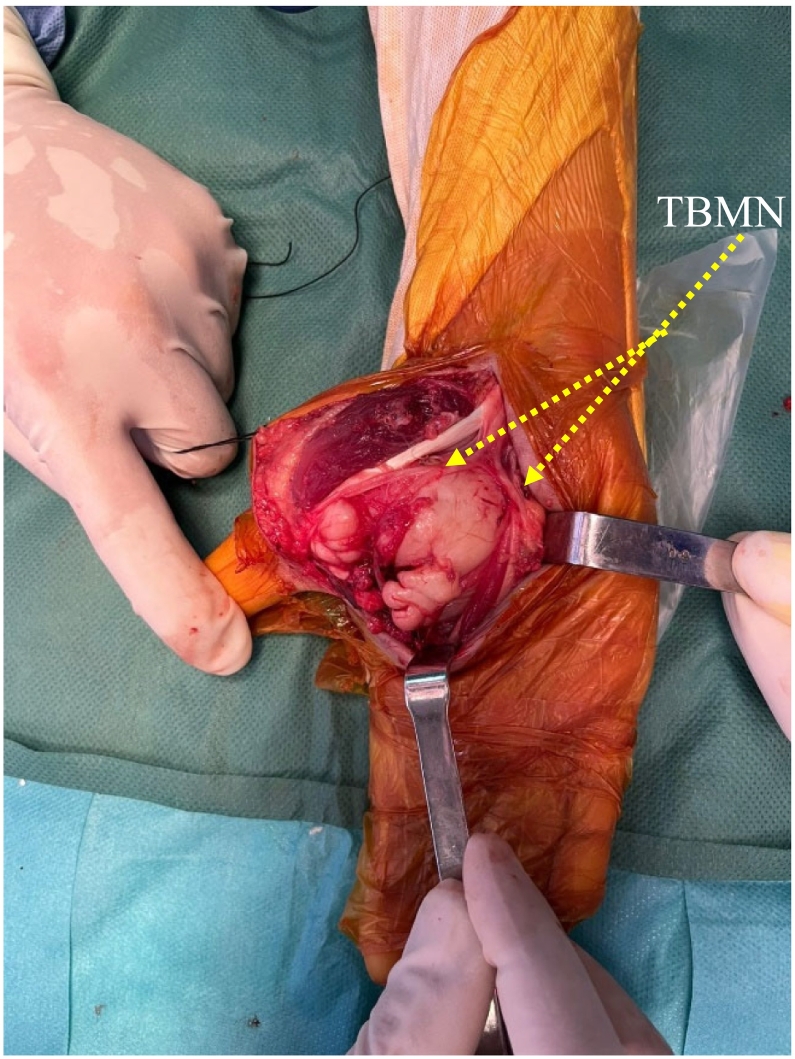
Intraoperative image: tumor displacing terminal branches of the median nerve (TBMN).

**Fig. 6:**
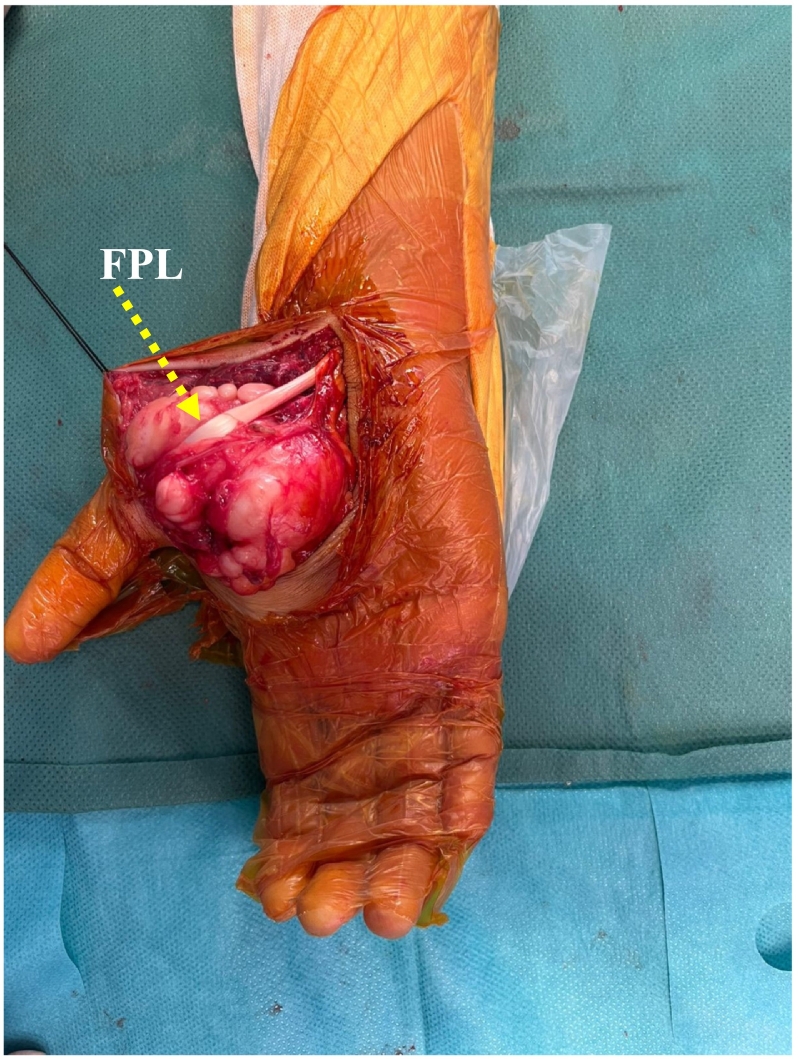
Intraoperative Image: The mass displacing the thenar muscles and compressing the flexor pollicis longus (FPL) without infiltration.

A biopsy was performed, which indicated a neurofibroma. Surgical excision of the tumor was carried out under axillary plexus block, with a pneumatic tourniquet applied at the root of the limb. A longitudinal incision following the thumb opposition crease was made (Fig. 4). Intraoperatively, we found a well-circumscribed, pearly white, multilobulated tumor closely adhering to and displacing the terminal branches of the median nerve (Fig. 5). The mass displaced the thenar muscles without infiltrating them and compressed the superficial and deep flexor tendons of the fingers medially, and the flexor pollicis longus tendon laterally (Fig. 6). Care was taken to preserve all vascular structures, including the superficial and deep palmar arches as well as the princeps pollicis artery, without sacrificing any branches. The tumor was easily separable, and a complete excision was achieved while preserving all anatomical structures, particularly the terminal branches of the median nerve. Skin closure was performed using separate sutures and a drain was placed. The postoperative recovery progressed smoothly, with no complications observed.

Histological analysis of the excised mass, which measured 8 × 6 × 3.5 cm and weighed 64 g, confirmed the diagnosis of a neurofibroma with no signs of malignancy.

**Fig. 7:**
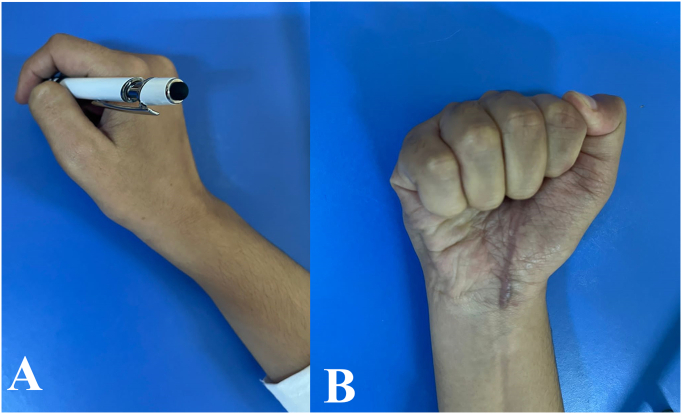
(A + B): postoperative recovery, with no nerve complications and full restoration of hand function.

The patient's postoperative recovery was excellent, with no nerve complications and a full restoration of hand function, including complete opposition, dexterity, and a full range of motion. At six weeks postoperatively, electroneuromyography was performed, revealing good conduction in both sensory and motor nerves, which confirmed the functional integrity of the median nerve. Additionally, hand function was objectively evaluated using the QuickDASH questionnaire [4], which yielded a score of 6, indicative of excellent recovery outcomes. (Fig. 7).

## 3 Discussion

Neurofibromas in the hand are exceedingly rare, particularly when located in the **thenar region**, which is a critical area for hand function. As summarized in Table 1, only a limited number of cases involving neurofibromas in the hand, particularly in the thenar region, have been reported in the literature, underscoring the rarity of this condition Solitary neurofibromas in the hand account for less than 1 % of cases [5], and those in the thenar region are even more uncommon. Our case involved a 23-year-old female presenting with a progressively enlarging mass in the thenar region over three years, accompanied by nocturnal pain without neurological deficits. In contrast to other hand neurofibromas, which are often painless and slow-growing [6], our patient's mass grew relatively quickly and caused significant discomfort, a finding that underscores the critical functional importance of the tumor's location.

**Table 1:** Comparative analysis of reported cases of hand neurofibromas.

Cases	Clinical data	Location	Imaging(MRI)	Surgery	Histology	Follow-up (months)
Our case	23y/o, 7 cm mass, nocturnal pain, no NF1	Thenar region	well-defined, T1 intermediate, STIR hyperintense	Complete excision, preserved median nerve	Benign, no malignancy	18 m, no recurrence
Bayomi et al. (2024) [6]	70 y/o, mass for 9 years, mild tingling	Palm	unavailable	Excision with neurotization of digital nerve	Neurofibroma, spindle cells, benign	7 m, no recurrence
Dogra et al. (2016) [5]	37 y/o, 6.5 cm mass, multiple NF1 signs	Digital nerves of the palm	well-defined, T1 isointense, T2 hyperintense	Complete excision of plexiform neurofibroma	Plexiform neurofibroma, spindle cells, benign	3 m, no recurrence
Reddy et al. (2007) [10]	44 y/o, large, recurrent mass in palm, tingling	Palm	heterogeneous signal, well-defined lobulated mass	Complete excision, some nerve damage	Neurofibroma, benign	6 m, persistent tingling, no recurrence
Kim et al. (2021) [7]	60y/o, 10 year mass in thumb, slow growth	Thumb	soft tissue mass, well-defined	Complete excision	Solitary neurofibroma, benign	14 m, no recurrence

Bayomi et al. [6] described a neurofibroma located in the **palm**, while Dogra et al. [5] reported a case in the **digital nerves** of the palm, both locations less critical for fine motor function than the thenar region. In our case, the tumor's location in the thenar eminence, which houses important muscles for thumb opposition, makes it particularly significant. The rapid onset of functional impairment in our patient, compared to the slower progression of symptoms in cases report by Bayomi et al. [6], highlights the clinical importance of location. Our case is further distinguished by the absence of systemic signs of neurofibromatosis, unlike the case reported by Dogra et al. [5], where the patient exhibited multiple NF1-related symptoms.

MRI findings in neurofibromas are generally consistent across cases, showing well-circumscribed masses with intermediate T1 and hyperintense T2 signals. In our case, the mass measured 54 × 55 × 33.4 mm in the **thenar region**, making it one of the largest solitary neurofibromas reported in the hand [7]. Most other reports involve smaller Neurofibromas, [7], where the mass measured only 3 cm and was located in the **thumb**. The size and thenar location in our case add to the rarity and clinical importance, as tumors in this region pose a greater risk of impairing thumb function and hand dexterity. Carvalho et al. [8] highlighted similar challenges in managing large solitary neurofibromas in critical locations, emphasizing the importance of surgical precision to avoid damage to vital structure.

Surgical excision is the preferred treatment for neurofibromas, especially when the tumor threatens motor or sensory functions. The challenge in our case was to carefully excise the tumor without damaging the terminal branches of the median nerve, which are crucial for thumb opposition. This approach was successful, as no functional deficits were noted postoperatively. In contrast, Dogra et al. [5] reported difficulty in excising a plexiform neurofibroma involving the **digital nerves** of the palm, where the diffuse nature of the tumor posed a higher risk for incomplete resection. This comparison underscores the relative surgical complexity of our case due to the tumor's **thenar location**, but the outcome was favorable with no compromise in function.

Histopathological analysis confirmed a benign neurofibroma in our case, composed of spindle cells and a myxoid stroma. No signs of malignancy were present, consistent with other reports of benign solitary neurofibromas [2,9]. Despite its large size and critical location, the tumor showed no atypical features. In contrast, plexiform neurofibromas, which are more common in NF1 cases like that described by Dogra et al. [5], tend to have a higher risk of recurrence and malignant transformation. The surgical outcomes summarized in Table 1 reveal that complete excision typically results in low recurrence rates, as demonstrated in our case. In comparison, Bayomi et al. [6] reported a patient with persistent sensory deficits after excision of a **palm neurofibroma**, and Dogra et al. [5] achieved short-term follow-up of 3 months without recurrence in a plexiform case. Our case is unique in that the tumor's large size and location in the thenar region, a critical area for hand function, posed significant risks. However, the outcome was excellent, with functional recovery objectively validated by a QuickDASH score of 6, [4] and no recurrence observed during the follow-up period.

**Fig. 8:**
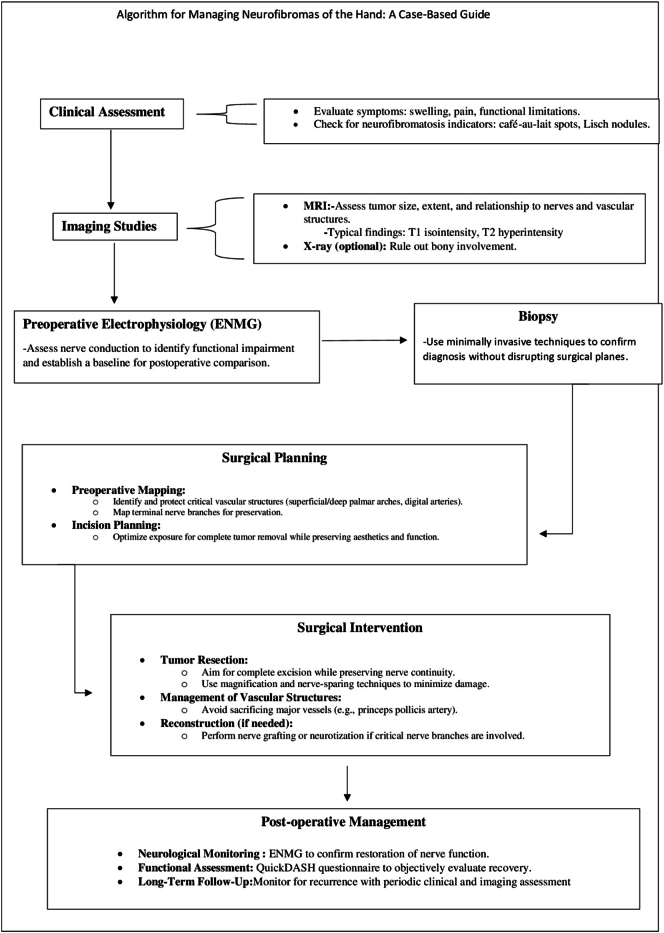
Algorithm for managing neurofibromas of the hand: a case-based guide.

To address the challenges of managing rare neurofibromas in critical hand locations, we propose a stepwise algorithm emphasizing detailed assessment, imaging, and electrophysiological testing to guide nerve-sparing surgical planning. The algorithm prioritizes complete excision using magnification to preserve structures, with postoperative monitoring and functional assessment, to ensure optimal outcomes and detect recurrence (Fig. 8).

## 4 Conclusion

This case of a large neurofibroma in the thenar region highlights the critical importance of early diagnosis and individualized surgical strategies in achieving favorable outcomes. Despite the tumor's size and challenging location, complete excision was achieved while preserving motor and sensory functions. The patient's full functional recovery and absence of recurrence after 18 months demonstrate the potential for excellent outcomes with meticulous surgical planning. However, as this report represents a single case, further studies are needed to generalize these findings and optimize management strategies for similar cases.

## Sources funding

No funding was sought for this study.

## Consent

Written informed consent was obtained from the patient for publication and any accompanying images. A copy of the written consent is available for review by the Editor-in-Chief of this journal on request.

## Ethical approval

Ethical approval was not necessary from the institute for case report.

## Guarantor

The guarantors for this case series are the first author and the corresponding author doctor ELMEHDI BACHKIRA.

## Declaration of competing interest

No benefits in any form have been received or will be received related directly or indirectly to the subject of this article.

## Figures and Tables

**Table:** 

SCARE Checklist			
Topic	Item	Checklist item description	Page number
**Title**	**1**	The words “case report” and the area of focus should appear in the title (e.g. presentation, diagnosis, surgical technique or device or outcome).	1
**Key Words**	**2**	3 to 6 key words that identify areas covered in this case report (include “case report” as one of the keywords).	1
**Abstract**	**3a**	Introduction—What is unique or educational about the case? What does it add to the surgical literature? Why is this important?	1
	**3b**	The patient's main concerns and important clinical findings.	
	**3c**	The main diagnoses, therapeutics interventions, and outcomes.	
	**3d**	Conclusion — what are the “take-away” lessons from this case?	
**Introduction**	**4**	A summary of why this case is unique or educational with reference to the relevant surgical literature and current standard of care (with references, 1–2 paragraphs). Nature of the institution in which the patient was managed; academic, community or private practice setting?	1
**Patient Information**	**5a**	De-identified demographic and other patient specific information including age, sex, ethnicity, occupation and other useful pertinent information e.g. BMI and hand dominance.	**2**
	**5b**	Presentation including presenting complaint and symptoms of the patient as well as the mode of presentation e.g. brought in by ambulance or walked into Emergency room or referred by family physician.	
	**5c**	Past medical and surgical history and relevant outcomes from interventions	
	**5d**	Drug history, family history including any relevant genetic information, and psychosocial history including smoking status and where relevant accommodation type, walking aids, etc.	
**Clinical Findings**	**6**	Describe the relevant physical examination and other significant clinical findings (include clinical photographs where relevant and where consent has been given).	2
**Timeline**	**7**	Inclusion of data which allows readers to establish the sequence and order of events in the patient's history and presentation (using a table or figure if this helps). Delay from presentation to intervention should be reported.	2
**Diagnostic Assessment**	**8a**	Diagnostic methods (physical exam, laboratory testing, radiological imaging, histopathology etc.).	2
	**8b**	Diagnostic challenges (access, financial, cultural).	
	**8c**	Diagnostic reasoning including other diagnoses considered	
	**8d**	Prognostic characteristics when applicable (e.g. tumor staging). Include relevant radiological or histopathological images in this section (the latter may sometimes be better placed in section 9).	
**Therapeutic Intervention**	**9a**	Pre-intervention considerations e.g. Patient optimisation: measures taken prior to surgery or other intervention e.g. treating hypothermia/hypovolaemia/hypotension in a burns patient, ICU care for sepsis, dealing with anticoagulation/other medications, etc.	2
	**9b**	Types of intervention(s) deployed and reasoning behind treatment offered (pharmacologic, surgical, physiotherapy, psychological, preventive) and concurrent treatments (antibiotics, analgesia, anti-emetics, nil by mouth, VTE prophylaxis, etc.). Medical devices should have manufacturer and model specifically mentioned.	
	**9c**	Peri-intervention considerations - administration of intervention (what, where, when and how was it done, including for surgery; anaesthesia, patient position, use of tourniquet and other relevant equipment, prep used, sutures, devices, surgical stage (1 or 2 stage, etc.). Pharmacological therapies should include formulation, dosage, strength, route, duration, etc.).	
	**9d**	Who performed the procedure - operator experience (position on the learning curve for the technique if established, specialisation and prior relevant training).	
	**9e**	Any changes in the interventions with rationale. Include intra-operative photographs and/or video or relevant histopathology in this section. Degree of novelty for a surgical technique/device should be mentioned e.g. “first in-human”.	
	**9f**	Post-intervention considerations e.g. post-operative instructions and place of care.	
**Follow-up and Outcomes**	**10a**	Clinician assessed and patient-reported outcomes (when appropriate) should be stated with inclusion of the time periods at which assessed. Relevant photographs/radiological images should provide e.g. 12 month follow-up.	2
	**10b**	Important follow-up measures - diagnostic and other test results. Future surveillance requirements - e.g. imaging surveillance of endovascular aneurysm repair (EVAR) or clinical exam/ultrasound of regional lymph nodes for skin cancer.	
	**10c**	Where relevant - intervention adherence and tolerability (how was this assessed).	
	**10d**	Complications and adverse or unanticipated events. Described in detail and ideally categorized in accordance with the Clavien-Dindo Classification. How they were prevented, diagnosed and managed. Blood loss, operative time, wound complications, re-exploration/revision surgery, 30-day post-op and long-term morbidity/mortality may need to be specified.	
**Discussion**	**11a**	Strengths, weaknesses and limitations in your approach to this case. For new techniques or implants - contraindications and alternatives, potential risks and possible complications if applied to a larger population. If relevant, has the case been reported to the relevant national agency or pharmaceutical company (e.g. an adverse reaction to a device).	3 + 4
	**11b**	Discussion of the relevant literature, implications for clinical practice guidelines and any relevant hypothesis generation.	
	**11c**	The rationale for your conclusions.	
	**11d**	The primary “take-away” lessons from this case report.	
**Patient Perspective**	**12**	When appropriate the patient should share their perspective on the treatments they received.	4
**Informed Consent**	**13**	Did the patient give informed consent for publication? Please provide if requested by the journal/editor. If not given by the patient, explain why e.g. death of patient and consent provided by next of kin or if patient/family untraceable then document efforts to trace them and who within the hospital is acting as a guarantor of the case report.	4
**Additional Information**	**14**	Conflicts of Interest, sources of funding, institutional review board or ethical committee approval where required.	5
